# Application of Machine Learning Methods to Improve the Performance of Ultrasound in Head and Neck Oncology: A Literature Review

**DOI:** 10.3390/cancers14030665

**Published:** 2022-01-28

**Authors:** Celia R. DeJohn, Sydney R. Grant, Mukund Seshadri

**Affiliations:** 1 Center for Oral Oncology, Roswell Park Comprehensive Cancer Center, Buffalo, NY 14263, USA; Celia.DeJohn@roswellpark.org; 2 Cell Stress and Biophysical Oncology Program, Roswell Park Comprehensive Cancer Center, Buffalo, NY 14263, USA; Sydney.Grant@roswellpark.org; 3 Medical Physics Program, Department of Radiology, University at Buffalo—Jacobs School of Medicine and Biomedical Sciences, Buffalo, NY 14203, USA

**Keywords:** ultrasound, head and neck cancer, machine learning, radiomics, artificial intelligence

## Abstract

**Simple Summary:**

Ultrasound (US) is a non-invasive imaging method that is routinely utilized in head and neck cancer patients to assess the anatomic extent of tumors, nodal and non-nodal neck masses and for imaging the salivary glands. In this review, we summarize the present evidence on whether the application of machine learning (ML) methods can potentially improve the performance of US in head and neck cancer patients. We found that published clinical literature on ML methods applied to US datasets was limited but showed evidence of improved diagnostic and prognostic performance. However, a majority of these studies were based on retrospective evaluation and conducted at a single center with a limited number of datasets. The conduct of multi-center studies could help better validate the performance of ML-based US radiomics and facilitate the integration of these approaches into routine clinical practice.

**Abstract:**

Radiomics is a rapidly growing area of research within radiology that involves the extraction and modeling of high-dimensional quantitative imaging features using machine learning/artificial intelligence (ML/AI) methods. In this review, we describe the published clinical evidence on the application of ML methods to improve the performance of ultrasound (US) in head and neck oncology. A systematic search of electronic databases (MEDLINE, PubMed, clinicaltrials.gov) was conducted according to Preferred Reporting Items for Systematic Reviews and Meta-Analyses (PRISMA) guidelines. Of 15,080 initial articles identified, 34 studies were selected for in-depth analysis. Twenty-five out of 34 studies (74%) focused on the diagnostic application of US radiomics while 6 (18%) studies focused on response assessment and 3 (8%) studies utilized US radiomics for modeling normal tissue toxicity. Support vector machine (SVM) was the most commonly employed ML method (47%) followed by multivariate logistic regression (24%) and k-nearest neighbor analysis (21%). Only 11/34 (~32%) of the studies included an independent validation set. A majority of studies were retrospective in nature (76%) and based on single-center evaluation (85%) with variable numbers of patients (12–1609) and imaging datasets (32–1624). Despite these limitations, the application of ML methods resulted in improved diagnostic and prognostic performance of US highlighting the potential clinical utility of this approach.

## 1. Introduction

The term ‘head and neck cancer’ is used to describe a heterogeneous group of neoplasms that can arise in multiple sites within this anatomic region including the mucosal epithelium of the oral and nasal cavities, larynx, pharynx, thyroid, and salivary glands [1]. Ultrasound (US) is a noninvasive imaging modality that is routinely employed in head and neck cancer patients to determine the anatomic extent and vascularity of tumors and nodal masses [2,3,4]. US methods are also utilized for diagnostic evaluation of suspicious thyroid nodules [5], and the differential diagnosis of salivary gland neoplasms [6]. These applications rely on the ability of US to detect changes in size, shape, margins, structure (e.g., echogenicity, presence of calcifications), and vascularity (blood flow and velocity). Although useful, these imaging characteristics measured from US images are limited in number and to a large extent, ignore the wealth of information that is captured within the individual pixels (2D) or voxels (3D) that make up the image.

An alternative approach that overcomes these limitations is “radiomics” and refers to the extraction and modeling of multi-dimensional quantitative descriptors (textural features) from imaging datasets [7]. In radiomics, textural features are calculated based on the shape, distribution of voxel intensity, and spatial relationships between neighboring voxels and subsequently modeled through statistical algorithms (“machine learning”). Given this ability of radiomics to extract quantitative, high-throughput, multi-dimensional information, it is assumed that such radiomic “phenotypes” may better capture the heterogeneity within the tumor microenvironment and as a consequence, exhibit improved performance for diagnostic and prognostic applications compared to traditional radiologic or clinical assessment criteria used in head and neck oncology [8].

In this review, we summarize the published evidence on the clinical application of US radiomics in head and neck oncology. We describe the radiomic approaches employed, the quality of the evidence presented, and the performance of these methods for the intended application. The translational barriers and potential solutions for the successful integration of radiomics into routine clinical practice in head and neck oncology are discussed.

## 2. Materials and Methods

### 2.1. Literature Search Strategy

A review of the literature was performed following the Preferred Reporting Items for Systematic Reviews and Meta-Analysis (PRISMA) guidelines [9]. A database search was conducted in MEDLINE/PubMed (National Center for Biotechnology Information, NCBI) and clinicaltrials.gov, ending on 31 March 2021. Databases were searched for the following terms: “head and neck cancer”, OR “oral cancer,” OR “tongue cancer”, OR “thyroid cancer”, OR “lymph node”, OR “salivary gland”, OR “parotid gland,” AND “ultrasound,” AND “radiomics” OR “texture analysis”.

### 2.2. Study Screening and Selection Criteria

The principal inclusion criteria were studies utilizing radiomics or textural analysis to analyze US datasets in head and neck cancer patients. Conference abstracts, reviews, and articles not focused on head and neck cancer and studies that did not employ US were excluded. Duplicate articles were removed. Articles not published in the last 10 years, not written in English, or focused on the application of radiomic methods to non-imaging data (e.g., genomic or histopathologic data) were also removed. Finally, studies that utilized deep learning techniques, such as neural networks, were excluded from our analysis.

### 2.3. Data Extraction and Reporting

All relevant data from the selected articles were extracted and tabulated into an Excel database (Microsoft Corporation, Washington, DC, USA). The following information was gathered from the studies and stratified in the following manner:

- Publication details: Data of publication and authorship information.
- Proposed clinical application: Diagnostic, prognostic, or toxicity assessment.
- Patient population: Anatomic site and type of head and neck cancer including primary tumor, lymph nodes.
- Study details: Study type (retrospective vs. prospective, single-center vs. multi-center), sample sizes (number of patients, number of datasets, testing/independent validation), treatment information, and outcome measures.
- Methodology: US instrumentation and probe frequency, radiomics platform employed (software and type of algorithm), statistical methodology, and performance metrics reported.

Graphical display of the data was performed using GraphPad Prism version 9.0.0 for Windows (GraphPad Software, San Diego, CA, USA).

### 2.4. Radiomics Quality Score (RQS)

The RQS is based on 16 criteria that encompass multiple domains for the evaluation of the quality of radiomic studies [10]. Briefly, these criteria include image protocol quality, segmentation, feature selection, clinical or biological correlates, statistical methods and performance metrics, validation, comparison to the gold standard, cost-effectiveness, and open science. The RQS uses a point-based system to award or penalize points depending on the fulfillment of the criteria with a maximum score of 36 (100%). Training, validation, and test sets were defined as follows: (1) a training dataset is a sample of data used to fit the model, (2) a validation set is a cohort that provides an unbiased evaluation of a model fit on the training set and (3) a test set is an external or independent sample of data that should only be reported for the algorithm once it has been trained and validated [11,12].

## 3. Results

### 3.1. Search Results

**Figure 1:**
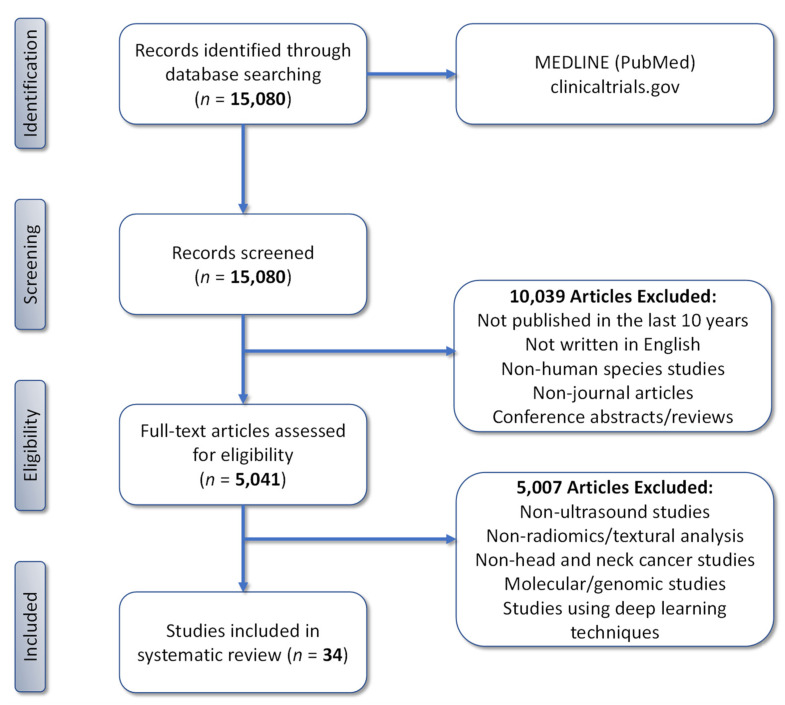
PRISMA flowchart (adapted from PRISMA group, 2020) describing the study selection process.

The initial search of the electronic databases retrieved 15,080 candidate articles that were screened for eligibility and subsequent evaluation. Application of our pre-defined criteria resulted in the exclusion of 10,039 articles. The remaining 5041 articles underwent a comprehensive assessment based on a full-text examination to confirm eligibility. Following a second screen, an additional 5007 articles were excluded based on the imaging modality studied, lack of radiomic analysis of image datasets, cancer sites, molecular or genomic studies, and studies that utilized deep learning methods. This resulted in a final total of 34 articles that were included for in-depth assessment (Figure 1).

### 3.2. Clinical Applications of US Radiomics in Head and Neck Oncology

**Figure 2:**
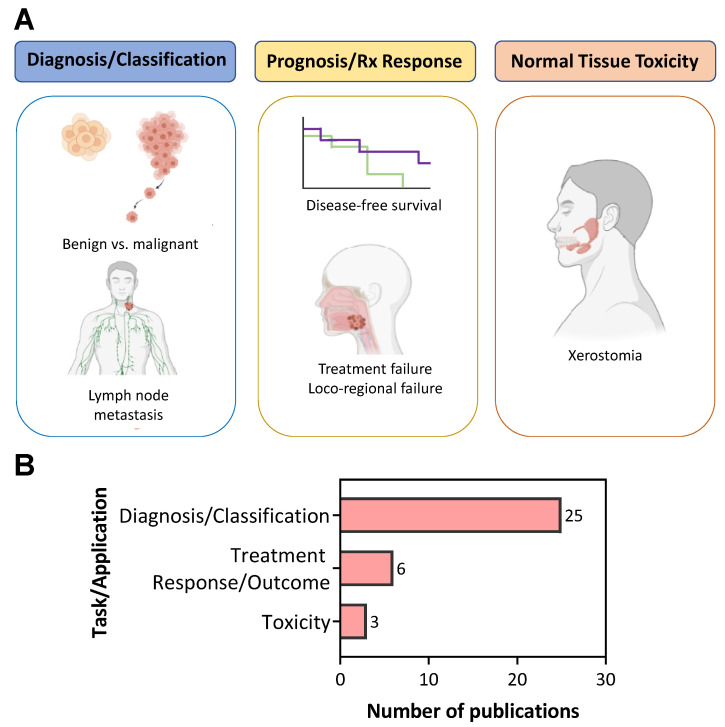
Current applications of US radiomics in head and neck oncology. (**A**) Diagnostic applications (left) based on histopathologic findings for the differentiation of benign and malignant thyroid nodules, as well as the classification of lymph node metastasis. Prognostic applications (middle) based on patient outcomes, such as disease-free survival, or response to treatment (Rx response), such as locoregional recurrence. Prediction of treatment toxicity (right) due to salivary gland injury from radiation therapy. (**B**) Number of publications grouped by clinical task or application.

Figure 2A summarizes the three published clinical applications of US radiomics in head and neck cancer patients. A majority of the studies (*n* = 25/34; 74%) were focused on evaluating the potential of US radiomics for disease diagnosis (Figure 2B). Of these studies, 56% examined the utility of US radiomics to distinguish benign and malignant thyroid nodules (*n* = 14/25) and 44% were focused on the identification of metastatic lymph nodes (*n* = 11/25). The second clinical application of US radiomics was to prognosticate response to standard of care chemotherapy or radiation (*n* = 6/34, 18%). A relatively small number of studies (*n* = 3/34, 8%) examined the ability of US radiomics to assess toxicity, specifically, to predict xerostomia (severe dry mouth) following radiation injury to salivary glands in head and neck cancer patients.

### 3.3. Characteristics of US Radiomics Studies in Head and Neck Cancer

**Figure 3:**
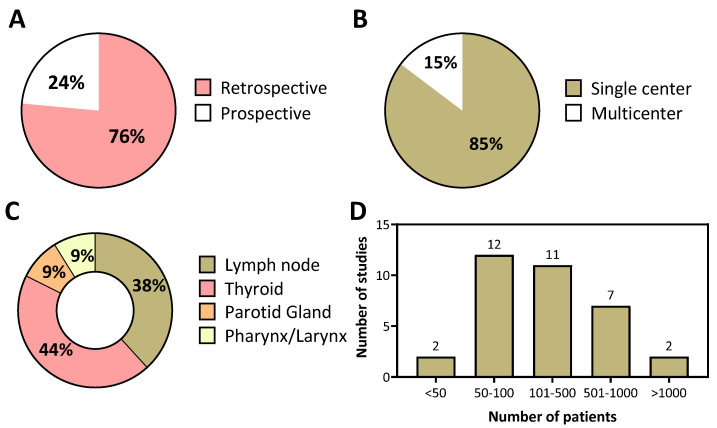
Design characteristics and sample sizes employed in US radiomics studies in head and neck cancer. (**A**) A majority of the published studies of US radiomics employed a retrospective design and (**B**) were conducted at a single center. (**C**) The thyroid glands represented the most common site imaged in these studies followed by the lymph nodes. (**D**) The number of patients evaluated in these published studies exhibited a wide range (12–1609) with a majority of studies (~60%) reporting analysis of data from over 100 patients.

The data presented in Figure 3 summarizes the design characteristics and head and neck cancer sites of US radiomics studies reported in the literature. A majority of the studies were based on retrospective analysis (*n* = 26, 76%; Figure 3A) and conducted at a single center (*n* = 29, 85%; Figure 3B). In clinical practice, US is predominantly used for the evaluation and staging of superficial structures in the head and neck region. Therefore, not so surprisingly, a large proportion of published studies were focused on the examination of the thyroid (*n* = 15, 44%) or lymph nodes (*n* = 13, 38%) (Figure 3C). A relatively small number of studies (<10%) utilized US radiomics to study the salivary glands and primary tumors in the pharynx and larynx. The number of patients evaluated in these studies varied widely from 12 to 1609 (median value 137) with a majority of studies reporting findings based on analysis of data from over 100 patients (Figure 3D).

### 3.4. Imaging Datasets in US Radiomics Studies

**Figure 4:**
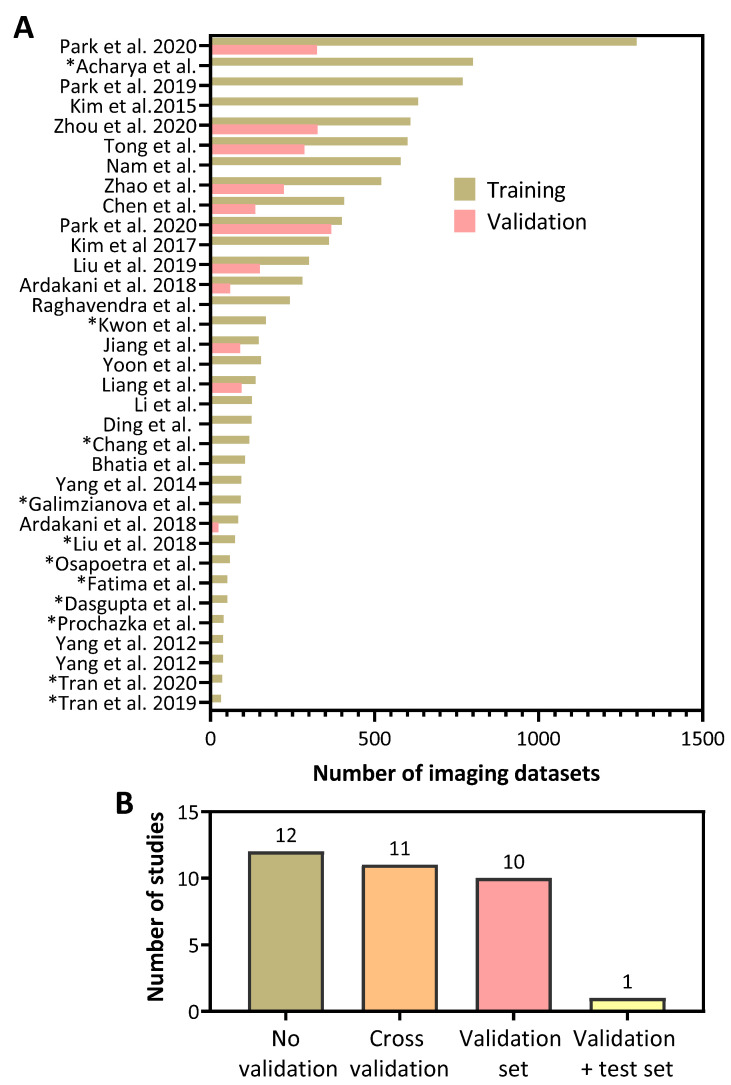
Number of images used in training and validation of US radiomic model performance in head and neck cancer. (**A**) Number of images used in the training and validation sets in published studies of US radiomics in head and neck cancer patients. (**B**) Number of studies that employed validation and test sets to evaluate model performance. Asterisk (*) indicates studies that did not have an independent validation set but did include cross-validation as part of their analyses.

It is well recognized that the size of the imaging datasets used for training (i.e., number of datasets used to develop the model) critically influences the performance of ML models. Subsequent examination of the performance of developed ML models using an independent validation dataset (a cohort that provides an unbiased evaluation of a model) is also essential to assess their true utility. And finally, a test set is an external or independent sample of data that is used to report the performance of the algorithm once it has been trained and validated [11,12]. We, therefore, examined the number of imaging datasets employed for training and validation in published US radiomics studies [13,14,15,16,17,18,19,20,21,22,23,24,25,26,27,28,29,30,31,32,33,34,35,36,37,38,39,40,41,42,43,44,45,46] (Figure 4A). This analysis revealed a considerable degree of variability in the number of images used in the training (range 32–1299) datasets for developing the ML models. Notably, less than half of the studies included an independent validation dataset (*n* = 11, 32%). The majority of studies (Figure 4B) did not include a validation method to evaluate the performance of their model (*n* =12, 35%), or included cross-validation in their analyses (*n* = 11, 32%), Only a single study employed a validation and an independent test set. In studies that utilized a validation set, the number of images used in these validation datasets was considerably lower than the number of images in the training dataset (range 0 to 368). An asterisk (*) in front of a study name indicates that the authors used cross-validation as a means of validating their training model (Figure 4A).

### 3.5. Machine Learning Methods Employed in US Radiomics Studies

**Figure 5:**
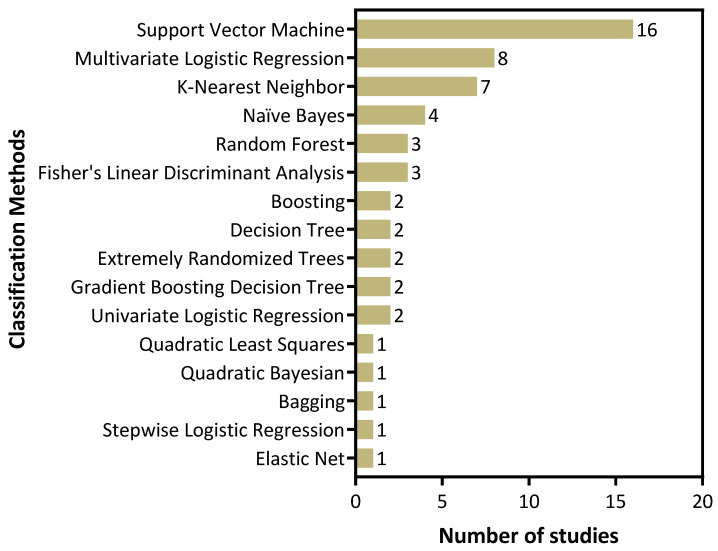
Machine learning methods used in US radiomics studies of head and neck cancer. The most commonly employed ML technique for classification was support vector machine (SVM) followed by multivariate logistic regression and k-nearest neighbor (kNN).

Next, we examined the ML methods employed in published US radiomic studies independent of the clinical application or the outcome measures studied (Figure 5). This analysis revealed that several studies employed more than one ML method in their analyses. The most commonly employed ML technique for classification was support vector machine (SVM) followed by multivariate logistic regression and k-nearest neighbor (kNN).

### 3.6. Reporting Completeness and Methodologic Quality Appraisal

We assessed the reporting completeness and the quality of the methodological aspects of model development and validation using the RQS [10] (Supplementary Table S1, [13,14,15,16,17,18,19,20,21,22,23,24,25,26,27,28,29,30,31,32,33,34,35,36,37,38,39,40,41,42,43,44,45,46]). Nearly all of the publications included an adequate description regarding the imaging protocol (at minimum, the type of transducer, and the central frequency). Additionally, most of the studies were awarded points for including discrimination, model performance, statistics, as well as feature reduction methods. However, over half of the studies (*n* = 21, 62%) lacked a robust description of segmentation methods. Additionally, many of the studies did not include multivariate analysis of radiomic features with non-radiomic features, such as clinical or histologic data (*n* = 19, 56%). Studies were not penalized for using cross-validation to corroborate their training set, but 5 points were deducted if there was no validation method at all (*n* = 12, 35%). Surprisingly, 94% of studies (*n* = 32) did not provide/use open-source codes/software.

### 3.7. Performance of US Radiomic Models for Diagnostic Applications in Head and Neck Oncology

As stated earlier, the principal diagnostic applications of US radiomics in head and neck oncology include the classification of benign and malignant thyroid nodules and the diagnosis of lymph node metastases. Table 1 summarizes the radiomics approach, methodology and the principal findings of 25 studies focused on the evaluation of US radiomics for diagnostic applications in head and neck oncology. A high proportion of studies (23/25) examining the ability of US radiomics to predict thyroid malignancy or lymph node metastases in head and neck cancer patients achieved an AUC or c-index above 0.7. Most of the ML models that were developed achieved the highest diagnostic performance when combining clinical and radiomic features. Additionally, most of the authors compared their models to the current clinical paradigms or “gold standard” method to demonstrate the added value of radiomics.

Park et al. [31] developed an US radiomics model to differentiate benign and malignant thyroid nodules and investigated its potential as a diagnostic adjunct to improve the performance of existing risk stratification guidelines. This retrospective study included a training set (*n* = 1299) and a validation set (*n* = 325). Thyroid nodules were manually segmented on US images and radiomic features were extracted using an in-house developed algorithm in MATLAB 2018b (The MathWorks, Inc., Natick, Massachusetts, USA). Based on the training set, the least absolute shrinkage and selection operator (LASSO) feature selection method reduced the number of features from 730 to 66. A radiomics score for each nodule was calculated to reflect the risk of malignancy. The performances of the American College of Radiology (ACR) and American Thyroid Association (ATA) guidelines were compared with the combined diagnostic performance of the guidelines and the radiomics score. When ACR or ATA guidelines were combined with the radiomics score (using a 5% predicted malignant risk cutoff, Rad_5%), radiologists showed an increased specificity (72.8%, *p* < 0.001), accuracy (73.2%, *p* < 0.001) and positive predictive value (42.1%, *p* < 0.001) with decreased unnecessary fine needle aspiration (FNA) biopsy rates (57.9%, *p* < 0.001) and no change in sensitivity (75.0%, *p* > 0.99). This study highlights the potential for US radiomics to act as a complementary tool that could assist in the discrimination of benign and malignant thyroid nodules.

Histopathologic evidence of lymph node involvement is critical in surgical planning and determining the extent of neck dissection in head and neck cancer patients. Depending on the number of lymph nodes and the levels of involved lymph nodes on imaging, advanced neck dissection techniques can enable the staging of the patient and potentially allow for less invasive approaches to managing tumor and nodal disease [47]. In this context, Jiang et al. [21] developed a nomogram based on multivariate logistic regression that incorporated US radiomic features and clinicopathologic characteristics of papillary thyroid carcinoma patients for the preoperative diagnosis of LNM. This retrospective study included a training cohort (*n* = 147) to build the model and a validation cohort (*n* = 90) to assess the performance of their model, collecting imaging data from two separate institutions. Nodules were manually delineated and 310 B-mode US radiomic features and 209 shear wave elastography (SWE) radiomic features were automatically extracted using the open-source software, Pyradiomics [48]. Then, a 2-step feature selection method was applied: a minimum redundancy maximum relevance (mRMR) algorithm was used to reduce the feature space and LASSO logistic regression method was applied to select the most meaningful features from the training dataset (final number of features = 6). Finally, an US radiomics nomogram was developed incorporating the radiomics score of the features and the independent clinical variables (e.g., multifocality). For comparison, an independent model was developed using the clinical variables alone. Their nomogram was built for the preoperative classification of LNM, which showed superior diagnostic performance compared to the clinical model in both the training (AUC of 0.851 vs. 0.800, *p* = 0.034) and validation datasets (AUC of 0.832 vs. 0.783, *p* = 0.048). Their nomogram showed that patients with a score greater than or equal to 0.574 were high-risk patients likely to have LNM (sensitivity of 86.84% and a positive predictive value of 70.21% in the validation cohort). This study presents the usefulness of US radiomics for the preoperative diagnosis of LNM, which has the potential to guide clinicians’ decisions in regard to performing FNA biopsies.

In another study, Li et al. [25] compared the clinical value of US characteristics of thyroid nodules to US radiomic features in diagnosing LNM prior to surgery. A total of 126 thyroid cancer patients with preoperative US images were retrospectively analyzed to predict LNM. Thyroid nodules were delineated by two radiologists and a total of 1079 radiomic features were extracted using closed-source software. Then, LASSO and principal component analysis (PCA) was used to reduce the multi-dimensional feature space to 91. Hypothesis testing and bagging were used to build the model based on these radiomic features. The diagnostic performance of their radiomics model was compared to the relationship between US characteristics and LNM, including shape, calcification, composition, and echogenicity. The analysis of US characteristics suggested that irregular shape and microcalcification were predictors of LNM in thyroid cancer patients (*p* = 0.025 and *p* = 0.004, respectively). However, ROC curve analysis for the diagnosis of LNM revealed that the radiomics model had markedly improved performance compared to US characteristics (AUC of 0.76 and 0.80 in the training and validation cohorts of the radiomics model respectively, vs. AUC of 0.591 and 0.629 for US characteristics of irregular shape and microcalcification, respectively). This study demonstrates the added value of radiomics analysis in screening meaningful US features in thyroid cancer patients for the preoperative prediction of LNM.

While several studies have demonstrated improved diagnostic performance with the use of ML methods, a small number of studies reported a lack of value addition with radiomics compared to traditional radiologic measures. Kim et al. [23] investigated optimal subsets of first- and second-order textural features (histogram and co-occurrence matrix features) for the discrimination of malignant and benign thyroid nodules in comparison to conventional radiologic characteristics (echogenicity, calcifications, shape). To evaluate the differences in both radiologic characteristics and textural features with malignant (*n* = 444) and benign (*n* = 189) thyroid nodules, confirmed by US-guided FNA, their diagnostic performance was assessed by calculating sensitivity, specificity, accuracy, positive predictive value (PPV), and negative predictive value (NPV). The authors reported that gray-scale ultrasound characteristics displayed the highest diagnostic performance, with 91% sensitivity, 91% specificity, 77% accuracy, 57% PPV, and 95% NPV. Among the first- and second-order textural features, the histogram parameter of mean intensity had the highest overall diagnostic performance, with 70% sensitivity, 65% specificity, 67% accuracy, 46% PPV, and 64% NPV. However, the overall performance of the mean intensity was significantly lower than that of gray-scale US characteristics (*p* < 0.001).

### 3.8. Performance of US Radiomics for Response Prediction in Head and Neck Cancer

The performance of US radiomics models for prognostic applications in predicting treatment response, locoregional recurrence, and treatment outcomes are summarized in Table 2. Dasgupta et al. [38] conducted a prospective study to investigate the role of US radiomics for the pretreatment prediction of recurrence in head and neck cancer patients (clinicaltrials.gov identifier NCT03908684). A total of 51 head and neck cancer patients with a primary site of oropharynx, hypopharynx, and larynx were enrolled in this study. Patients were treated with intensity-modulated radiation therapy or imaging-guided radiation therapy (70 Gy/33 fractions for 6–7 weeks), with or without concurrent chemotherapy. All patients were followed for a median of 38 months. For patients with confirmed recurrence, cross-sectional imaging and tissue diagnosis via biopsy were undertaken. Pretreatment US images were acquired up to 1 week prior to the start of radiation therapy and the largest lymph node was selected for imaging. Three different data modeling classifiers were built using a maximum of 3 features per model. The algorithms were tested using leave one out cross-validation (LOOCV). Recurrence (local, regional, or distant) served as the endpoint and was observed in 17 of the patients. The k-nearest neighbor (kNN) classifier demonstrated the highest sensitivity, specificity, and accuracy for predicting recurrence or no recurrence (76%, 71%, and 75%, respectively). This pilot clinical study is the first to demonstrate the potential clinical utility of US radiomics in head and neck cancer patients for the prediction of locoregional recurrence. Park et al. [41] developed an US radiomics signature to predict disease-free survival (DFS) in papillary thyroid carcinoma (PTC) patients and assessed the value of radiomics in comparison to clinicopathologic risk factors (including pathological tumor size and gross extrathyroidal extension). In their retrospective analysis, a total of 768 PTC patients that underwent preoperative US examination were enrolled. Tumors were manually segmented by a single radiologist and 730 candidate features were extracted using in-house radiomics analysis. The top 40 most useful radiomic features were selected using LASSO. A radiomics model integrating the clinicopathologic features improved the c-index compared to the clinical model alone (0.777 vs. 0.721, respectively) and demonstrated better performance in the estimation of DFS in PTC patients. While this study highlights the potential of US radiomics for risk stratification in patients with PTC, further validation is required on an independent dataset.

### 3.9. Performance of US Radiomics for Xerostomia Evaluation

Xerostomia (severe dry mouth) is one of the most common side effects of radiation therapy in head and neck cancer patients due to radiation damage to salivary glands [49]. Three studies shown in Table 3, create a model using features extracted from US images to distinguish between irradiated and healthy parotid glands. In a prospective study, Yang et al. [44] imaged the parotid glands of 12 patients post-RT and 7 healthy volunteers. Using an in-house developed MATLAB algorithm, they extracted 6 sonographic textural features. They observed that healthy volunteers showed tissue homogeneity and soft tissue echogenicity while irradiated glands showed greater heterogeneity. Significant differences were found in 4/6 sonographic features, all of which were found to have a *p*-value < 0.05. The most significant differences were found in areas of high intensity and high-intensity width. In a complementary analysis [45], Yang’s group also extracted gray-level co-occurrence matrix (GLCM) features from the parotid gland of 12 RT-treated patients and 7 healthy volunteers. This texture analysis again showed trends of heterogeneity in irradiated glands in addition to hyperechoic and hypoechoic lines and spots, decreased correlation, increased entropy, and lack of symmetry. Histological analysis performed on the parotid gland tissue provided a biological explanation for their radiomics analysis. While normal parotid glands show homogeneous regions of densely packed serous acinar cells post-radiation, many acini are lost leaving remaining cells that appear larger and more disorganized. In addition to patches of inflammatory infiltrates, these biological findings provide an explanation for heterogeneity in the radiation-damaged tissue. Additionally, post-radiation associated fibrosis may help explain hypoechoic spots and lines. In this prospective study, significant differences were observed for all 8 features of post-RT glands compared to normal (*p* < 0.05).

A larger follow-up retrospective study was also conducted by Yang et al. [46] where both parotid glands of 34 post-radiation patients and 13 healthy volunteers were imaged with US. RT-treated patients were further stratified into two groups, early toxicity occurring within the first 3 months of treatment completion and late toxicity occurring beyond 3 months of treatment completion. Extraction of echo-intensity histograms and sonographic features showed that 4/6 features achieved high diagnostic accuracy classifying acute toxicity vs. normal glands and late-toxicity vs. normal glands, while all parameters were useful in classifying patients, especially peak intensity, area under high-intensity portions of the curve and width of high-intensity portions of the histogram (AUC ≥ 0.90). The study demonstrated that radiomics could successfully differentiate between acute and late toxicity, while the radiologist participating in the study was unable to make the same distinction based on visual interpretation. These limited findings provide encouraging evidence on the potential of US radiomics in the assessment of radiation toxicity in head and neck cancer patients. It is important to recognize that although US is not routinely used in radiation treatment planning, radiomics using CT and MRI are being extensively investigated for their potential to improve the accuracy of tumor segmentation, dose-escalation, and prediction of tumor response to radiation therapy [50].

## 4. Discussion

This review summarizes the current literature on US radiomics in head and neck cancer. Our objectives were to evaluate and compare the textural and ML methodologies used for current US radiomic applications and to assess the added value of US radiomics for diagnostic and prognostic applications in head and neck cancer patients. 

US plays a key role in the clinical workup of head and neck cancer patients for diagnosis and staging. Traditionally, qualitative or semantic features observed on the images are used to describe tumor shape, size, and location. However, this conventional radiologic assessment is constrained by subjective scoring methods and limited sensitivity of the human eye to detect subtle variations in the signal. With radiomics, features that are mined from the images describe recurrent patterns or textures and the statistical inter-relationships between neighboring voxels quantitatively and objectively. 

The generation of robust and high-quality evidence on the performance of radiomics is critical for successful integration into clinical practice. Our review of the radiomics literature revealed that the majority of published studies of US radiomics are based on retrospective analysis of imaging datasets. Moreover, a majority of these studies were conducted at a single center with a limited number of datasets. To understand the strength of the published evidence on the potential utility of US radiomics in head and neck oncology, we used the RQS. The RQS has 16 main components that assess the rigor of the studies, from image acquisition to feature extraction/reduction, model validation, biological associations, and clinical significance. However, the majority of the studies included in this review received a score of less than 50%. Several systematic reviews investigating the utility of radiomics in breast and lung cancers have also reported low RQS (less than 50%), with a lack of external validation or prospective study [51,52,53]. Many of the studies in this review did not include a validation set, or only included cross-validation due to insufficient sample sizes. Validation of the model is an indispensable component of radiomics analysis and must not be left out. Validation techniques lend credibility to the trained model and assess its overall performance. A test set must only be implemented once a model is properly trained and validated. Finally, reporting open-access data and codes is vital to radiomics being accepted in both scientific and clinical communities.

The conduct of multi-center studies in the future could further validate the performance of ML-based US radiomics and facilitate the integration of these approaches into routine clinical practice. In this regard, it is important to consider the standardization of imaging methods using phantoms. The studies included in this review employed different US probe frequencies and therefore, resolution limits. Technical fluctuations within and between scanners, different imaging parameters, or radiologists can also substantially influence the variation of radiomics features. It is therefore imperative that such studies adhere to guidelines that have been developed including the Image Biomarker Standardization Initiative (IBSI) [54]. Clinical trials aimed at evaluating interventions using radiomic approaches should also adhere to recently developed AI guidelines to Standard Protocol Items: Recommendations for Interventional Trials (SPIRIT-AI) and Consolidated Standards of Reporting Trials (CONSORT-AI) [55]. Adherence to these standards will enable the generation of high-quality evidence on the performance of radiomics for specific clinical applications through structured data pipelines and facilitate the timely implementation of such pipelines for routine clinical use. Such efforts will likely require sustained engagement and partnership between academia and industry.

Finally, demonstrating the benefit or superior performance of radiomics over standard radiologic or clinical measures is critical to the widespread adoption of ML/AI. It is therefore important to recognize that not all clinical tasks require or may benefit from the use of radiomics. In this regard, recent studies have reported on the knowledge and attitude of radiologists towards the clinical adoption of AI [56,57,58]. A survey of over 1000 radiologists revealed that a majority (77%) of participants had a positive attitude and were favorable towards the adoption of AI. The perceived benefits of applying AI to routine radiologic practice reported by the participants included lower diagnostic error rates (73% of respondents) and optimization of radiologist workload (68% of respondents) while the risk of diminished professional reputation and increased costs, the higher workload for AI system maintenance and data analysis (39% of respondents) were the main concerns [56]. Similarly, a qualitative study by Chen et al. observed that radiologists believe that AI has the potential to assist in performing repetitive tasks which in turn could allow them to focus on challenging clinical scenarios or reads [57]. The study also showed that the awareness and knowledge about AI greatly varied among professionals in radiology. Consistent with this finding, a multi-institutional survey conducted with over 1000 radiologists and radiology residents found that limited AI-specific knowledge was associated with intimidation (OR 1.56, 95% CI 1.10–2.21, *p* = 0.01), while intermediate or advanced AI-specific knowledge was associated with optimism (OR 0.40, 95% CI 0.20–0.80, *p* = 0.01) [58]. Developing a standardized framework for the education and training of medical professionals and trainees on ML/AI methods is, therefore, critical [59]. While the overall attitude and perception of AI among radiologists and radiation oncologists are generally positive [55,56,57,58,60], there is skepticism among physicians in other specialties (e.g., surgeons) on the claims of AI and concerns regarding the high risk of bias and lack of transparency in published studies [61,62]. It is therefore important to engage all stakeholders including radiologists, medical and radiation oncologists, surgeons, and patients during the development of AI studies to ensure meaningful and successful adoption of AI-based approaches in the clinical setting.

## 5. Conclusions

The published body of evidence on US radiomics in head and neck oncology is limited. However, findings from clinical studies in the literature provide encouraging lines of evidence on the potential of ML methods to improve the diagnostic and prognostic performance of US in head and neck oncology.

## Figures and Tables

**Table 1:** Summary of reported diagnostic performance of US radiomics in head and neck oncology (studies have been summarized in alphabetical order).

Authors	Radiomics Platform	Number of Features	Statistical Analysis/Model Performance
Acharya et al., 2012 [13]	Not reported	5	Malignant vs. benign thyroid nodules Training AUC: 0.99
Ardakani et al., 2018 (Eur J Radiol) [14]	Not reported	40	Hot (hyperfunctioning) vs. cold (hypofunctioning) thyroid nodules Training AUC: 0.99 (95% CI: 0.978, 1.000) Validation AUC: 0.95 (95% CI: 0.874, 1.000)
Ardakani et al., 2018 (J Ultrasound Med) [15]	Not reported	4	LNM vs. no LNM Radiologic + textural features Training AUC: 0.99 (95% CI: 0.998, 0.999) Validation AUC: 0.95 (95% CI: 0.911, 0.993)
Bhatia et al., 2016 [16]	MATLAB	15	Malignant vs. benign thyroid nodules Training AUC: 0.97 (*p* < 0.0001)
Chang et al., 2016 [17]	Not reported	74	Malignant vs. benign thyroid nodules Adaboost CAD AUC: 0.98 RAD AUC: 0.98
Chen et al., 2020 [18]	MATLAB	23	Benign vs. lymphomatous AUC: 0.95 (*p* < 0.001) Lymphomatous vs. metastatic AUC: 0.93 (*p* < 0.001) Benign vs. malignant AUC: 0.84 (*p* < 0.001) Benign vs. metastatic AUC: 0.72 (*p* < 0.001)
Ding et al., 2012 [19]	Not reported	Not reported	Malignant vs. benign thyroid nodules Training accuracy: 95%
Galimzianova et al., 2020 [20]	Not reported	219	Malignant vs. benign thyroid nodules Training AUC: 0.83 (95% CI: 0.715, 0.942)
Jiang et al., 2020 [21]	PyRadiomics	6	LNM vs. no LNM Training AUC: 0.85 (95% CI: 0.791, 0.912) Validation AUC: 0.83 (95% CI: 0.749, 0.916)
Kim et al., 2015 [22]	MATLAB	10	Malignant vs. benign thyroid nodules Gray-scale AUC: 0.80 Elastography AUC: 0.68
Kim et al., 2017 [23]	MATLAB	5	LNM vs. no LNM OR: 0.98; 95% CI: 0.48-1.99, *p* > 0.05
Kwon et al., 2020 [24]	PyRadiomics	6	LNM vs. no LNM Training AUC: 0.93 Validation AUC: 0.90
Li et al., 2020 [25]	Ultrosomics	690	LNM vs. no LNM Training AUC: 0.76 Validation AUC: 0.80
Liang et al., 2018 [26]	AI Kit	19	Malignant vs. benign thyroid nodules Training AUC: 0.92 (95% CI: 0.877, 0.965) Validation AUC: 0.93 (95% CI: 0.884, 0.977)
Liu et al., 2018 [27]	MATLAB	25	LNM vs. no LNM (B-mode + SE-US) Training AUC: 0.90
Liu et al., 2019 [28]	MATLAB	50	LNM vs. no LNM Training AUC: 0.78 (95% CI: 0.731, 0.833) Validation AUC: 0.73 (95% CI: 0.653, 0.801)
Nam et al., 2016 [29]	MATLAB	5	Malignant vs. benign thyroid nodules Skewness AUC: 0.61 (95% CI: 0.563, 0.647) Kurtosis AUC: 0.65 (95% CI: 0.607, 0.691) Entropy AUC: 0.64 (95% CI: 0.596, 0.681)
Park et al., 2020 [30]	MATLAB	14	LNM vs. no LNM Training AUC: 0.71 (95% CI: 0.649, 0.770) Validation AUC: 0.62 (95% CI: 0.560, 0.682)
Park et al., 2021 [31]	MATLAB	66	Malignant vs. benign thyroid nodules Training AUC: 0.85 (95% CI: 0.830, 0.870) Validation AUC: 0.75 (95% CI: 0.690, 0.810)
Prochazka et al., 2019 [32]	MATLAB	Not reported	Malignant vs. benign thyroid nodules Accuracy: 94%
Raghavendra et al., 2017 [33]	Not reported	Not reported	Malignant vs. benign thyroid nodules Training AUC: 0.94
Tong et al., 2020 [34]	MATLAB	21	LNM vs. no LNM US radiomics nomogram Training AUC: 0.94 (95% CI: 0.911, 0.982) Validation AUC: 0.91 (95% CI: 0.842, 0.987)
Yoon et al., 2021 [35]	MATLAB	15	Malignant vs. benign thyroid nodules Radiomics score + clinical variables AUC: 0.84 (95% CI: 0.775, 0.897) Clinical variables alone AUC: 0.58 (95% CI: 0.435, 0.693)
Zhao et al., 2021 [36]	Intelligence Foundry	6	Malignant vs. benign thyroid nodules ML-assisted US visual approach Training AUC: Not reported Validation AUC: 0.90 Test AUC: 0.92
Zhou et al., 2020 [37]	MATLAB	23	LNM vs. no LNM Training AUC: 0.87 (95% CI: 0.802, 0.938) Validation AUC: 0.86 (95% CI: 0.785, 0.930)

AUC, area under the curve; LNM, lymph node metastasis; CAD AUC, computer-aided diagnosis area under the curve; RAD AUC, radiologist area under the curve.

**Table 2:** Summary of prognostic performance of US radiomics in head and neck oncology reported in the literature (studies have been summarized in alphabetical order).

Authors	Radiomics Platform	Number of Features	Statistical Analysis/Model Performance
Dasgupta et al., 2020 [38]	MATLAB	31	Recurrence vs. no recurrence kNN-based model a priori to treatment Training AUC: 0.74
Fatima et al., 2020 [39]	MATLAB	31	Recurrence vs. no recurrence Pre-treatment AUC: 0.71 1-week post-treatment AUC: 0.75 4 weeks post-treatment AUC: 0.81
Osapoetra et al., 2021 [40]	MATLAB	105	Prediction of clinical outcome (early responders vs. late responders vs. progressive disease) SVM AUC: 0.91
Park et al., 2019 [41]	MATLAB	40	Estimation of disease-free survival C-index: 0.78 (95% CI: 0.735, 0.829)
Tran et al., 2019 [42]	MATLAB	41	Complete vs. partial responders Univariate models kNN AUC: 0.81 (95% CI: 0.640, 0.980) naive-Bayes AUC: 0.87 (95% CI: 0.730, 1.010)
Tran et al., 2020 [43]	MATLAB	31	Complete vs. partial response Univariate kNN classifier 24 h post-RT AUC: 0.74 1-week post-RT AUC: 0.81 4 weeks post-RT AUC: 0.80

**Table 3:** Summary of treatment toxicity applications.

Authors	Radiomics Platform	Number of Features	Statistical Analysis/Model Performance
Yang et al., 2012 [44]	MATLAB	8	Significant differences observed for all 8 features of post-RT parotid glands compared to normal (*p* < 0.05)
Yang et al., 2012 [45]	MATLAB	6	Normal parotid gland R^2^ = 0.99 Irradiated parotid gland: R^2^ = 0.99
Yang et al., 2014 [46]	MATLAB	6	Acute toxicity vs. late toxicity Peak intensity AUC: 0.90 (*p* < 0.001)

## Supplementary Materials

The following are available online at https://www.mdpi.com/article/10.3390/cancers14030665/s1, Table S1: Performance of US radiomics methods for studies included in this review determined using radiomics quality score (RQS).

## References

[1] Chow L.Q.M. (2020). Head and Neck Cancer. N. Engl. J. Med..

[2] Klein Nulent T.J.W., Noorlag R., Van Cann E.M., Pameijer F.A., Willems S.M., Yesuratnam A., Rosenberg A.J.W.P., de Bree R., van Es R.J.J. (2018). Intraoral ultrasonography to measure tumor thickness of oral cancer: A systematic review and meta-analysis. Oral Oncol..

[3] Jayachandran S., Sachdeva S.K. (2012). Diagnostic accuracy of color doppler ultrasonography in evaluation of cervical lymph nodes in oral cancer patients. Indian J. Dent. Res..

[4] Dudau C., Hameed S., Gibson D., Muthu S., Sandison A., Eckersley R.J., Clarke P., Cosgrove D.O., Lim A.K. (2014). Can contrast-enhanced ultrasound distinguish malignant from reactive lymph nodes in patients with head and neck cancers?. Ultrasound Med. Biol..

[5] Brito J.P., Gionfriddo M.R., Al Nofal A., Boehmer K.R., Leppin A.L., Reading C., Callstrom M., Elraiyah T.A., Prokop L.J., Stan M.N. (2014). The accuracy of thyroid nodule ultrasound to predict thyroid cancer: Systematic review and meta-analysis. J. Clin. Endocrinol. Metab..

[6] Gritzmann N., Rettenbacher T., Hollerweger A., Macheiner P., Hübner E. (2003). Sonography of the salivary glands. Eur. Radiol..

[7] Liu Z., Wang S., Dong D., Wei J., Fang C., Zhou X., Sun K., Li L., Li B., Wang M. (2019). The applications of radiomics in precision diagnosis and treatment of oncology: Opportunities and challenges. Theranostics.

[8] Van Dijk L.V., Fuller C.D. (2021). Artificial Intelligence and Radiomics in Head and Neck Cancer Care: Opportunities, Mechanics, and Challenges. Am. Soc. Clin. Oncol. Educ. Book.

[9] Page M.J., McKenzie J.E., Bossuyt P.M., Boutron I., Hoffmann T.C., Mulrow C.D., Shamseer L., Tetzlaff J.M., Akl E.A., Brennan S.E. (2021). The PRISMA 2020 statement: An updated guideline for reporting systematic reviews. BMJ.

[10] Lambin P., Leijenaar R.T.H., Deist T.M., Peerlings J., de Jong E.E.C., van Timmeren J., Sanduleanu S., Larue R.T.H.M., Even A.J.G., Jochems A. (2017). Radiomics: The bridge between medical imaging and personalized medicine. Nat. Rev. Clin. Oncol..

[11] Debray T.P.A., Vergouwe Y., Koffijberg H., Nieboer D., Steyerberg E.W., Moons K.G.M. (2015). A new framework to enhance the interpretation of external validation studies of clinical prediction models. J. Clin. Epidemiol..

[12] Steyerberg E.W., Vickers A.J., Cook N.R., Gerds T., Gonen M., Obuchowski N., Pencina M.J., Kattan M.W. (2010). Assessing the performance of prediction models: A framework for traditional and novel measures. Epidemiology.

[13] Acharya U.R., Faust O., Sree S.V., Molinari F., Suri J.S. (2012). ThyroScreen system: High resolution ultrasound thyroid image characterization into benign and malignant classes using novel combination of texture and discrete wavelet transform. Comput. Methods Programs Biomed..

[14] Ardakani A.A., Mohammadzadeh A., Yaghoubi N., Ghaemmaghami Z., Reiazi R., Jafari A.H., Hekmat S., Shiran M.B., Bitarafan-Rajabi A. (2018). Predictive quantitative sonographic features on classification of hot and cold thyroid nodules. Eur. J. Radiol..

[15] Ardakani A.A., Reiazi R., Mohammadi A. (2018). A clinical decision support system using ultrasound textures and radiologic features to distinguish metastasis from tumor-free cervical lymph nodes in patients with papillary thyroid carcinoma. J. Ultrasound Med..

[16] Bhatia K.S.S., Lam A.C.L., Pang S.W.A., Wang D., Ahuja A.T. (2016). Feasibility study of texture analysis using ultrasound shear wave elastography to predict malignancy in thyroid nodules. Ultrasound Med. Biol..

[17] Chang Y., Paul A.K., Kim N., Baek J.H., Choi Y.J., Ha E.J., Lee K.D., Lee H.S., Shin D., Kim N. (2016). Computer-aided diagnosis for classifying benign versus malignant thyroid nodules based on ultrasound images: A comparison with radiologist-based assessments. Med. Phys..

[18] Chen Y., Jiang J., Shi J., Chang W., Shi J., Chen M., Zhang Q. (2020). Dual-mode ultrasound radiomics and intrinsic imaging phenotypes for diagnosis of lymph node lesions. Ann. Transl. Med..

[19] Ding J., Cheng H.D., Huang J., Zhang Y., Liu J. (2012). An improved quantitative measurement for thyroid cancer detection based on elastography. Eur. J. Radiol..

[20] Galimzianova A., Siebert S.M., Kamaya A., Rubin D.L., Desser T.S. (2020). Quantitative framework for risk stratification of thyroid nodules with ultrasound: A step toward automated triage of thyroid cancer. AJR Am. J. Roentgenol..

[21] Jiang M., Li C., Tang S., Lv W., Yi A., Wang B., Yu S., Cui X., Dietrich C.F. (2020). Nomogram Based on Shear-Wave Elastography Radiomics Can Improve Preoperative Cervical Lymph Node Staging for Papillary Thyroid Carcinoma. Thyroid.

[22] Kim S.-Y., Kim E.-K., Moon H.J., Yoon J.H., Kwak J.Y. (2015). Application of texture analysis in the differential diagnosis of benign and malignant thyroid nodules: Comparison with gray-scale ultrasound and elastography. AJR Am. J. Roentgenol..

[23] Kim S.Y., Lee E., Nam S.J., Kim E.K., Moon H.J., Yoon J.H., Han K.H., Kwak J.Y. (2017). Ultrasound texture analysis: Association with lymph node metastasis of papillary thyroid microcarcinoma. PLoS ONE.

[24] Kwon M.-R., Shin J.H., Park H., Cho H., Kim E., Hahn S.Y. (2020). Radiomics based on thyroid ultrasound can predict distant metastasis of follicular thyroid carcinoma. J. Clin. Med..

[25] Li F., Pan D., He Y., Wu Y., Peng J., Li J., Wang Y., Yang H., Chen J. (2020). Using ultrasound features and radiomics analysis to predict lymph node metastasis in patients with thyroid cancer. BMC Surg..

[26] Liang J., Huang X., Hu H., Liu Y., Zhou Q., Cao Q., Wang W., Liu B., Zheng Y., Li X. (2018). Predicting malignancy in thyroid nodules: Radiomics score versus 2017 American College of Radiology Thyroid Imaging, Reporting and Data System. Thyroid.

[27] Liu T., Ge X., Yu J., Guo Y., Wang Y., Wang W., Cui L. (2018). Comparison of the application of B-mode and strain elastography ultrasound in the estimation of lymph node metastasis of papillary thyroid carcinoma based on a radiomics approach. Int. J. Comput. Assist. Radiol. Surg..

[28] Liu T., Zhou S., Yu J., Guo Y., Wang Y., Zhou J., Chang C. (2019). Prediction of lymph node metastasis in patients with papillary thyroid carcinoma: A radiomics method based on preoperative ultrasound images. Technol. Cancer Res. Treat..

[29] Nam S.J., Yoo J., Lee H.S., Kim E.K., Moon H.J., Yoon J.H., Kwak J.Y. (2016). Quantitative evaluation for differentiating malignant and benign thyroid nodules using histogram analysis of grayscale sonograms. J. Ultrasound Med..

[30] Park V.Y., Han K., Kim H.J., Lee E., Youk J.H., Kim E.K., Moon H.J., Yoon J.H., Kwak J.Y. (2020). Radiomics signature for prediction of lateral lymph node metastasis in conventional papillary thyroid carcinoma. PLoS ONE.

[31] Park V.Y., Lee E., Lee H.S., Kim H.J., Yoon J., Son J., Song K., Moon H.J., Yoon J.H., Kim G.R. (2021). Combining radiomics with ultrasound-based risk stratification systems for thyroid nodules: An approach for improving performance. Eur. Radiol..

[32] Prochazka A., Gulati S., Holinka S., Smutek D. (2019). Classification of thyroid nodules in ultrasound images using direction-independent features extracted by two-threshold binary decomposition. Technol. Cancer Res. Treat..

[33] Raghavendra U., Acharya U.R., Gudigar A., Hong Tan J., Fujita H., Hagiwara Y., Molinari F., Kongmebhol P., Hoong Ng K. (2017). Fusion of spatial gray level dependency and fractal texture features for the characterization of thyroid lesions. Ultrasonics.

[34] Tong Y., Li J., Huang Y., Zhou J., Liu T., Guo Y., Yu J., Zhou S., Wang Y., Chang C. (2021). Ultrasound-based radiomic nomogram for predicting lateral cervical lymph node metastasis in papillary thyroid carcinoma. Acad. Radiol..

[35] Yoon J., Lee E., Kang S.-W., Han K., Park V.Y., Kwak J.Y. (2021). Implications of US radiomics signature for predicting malignancy in thyroid nodules with indeterminate cytology. Eur. Radiol..

[36] Zhao C.K., Ren T.T., Yin Y.F., Shi H., Wang H.X., Zhou B.Y., Wang X.R., Li X., Zhang Y.F., Liu C. (2021). A comparative analysis of two machine learning-based diagnostic patterns with Thyroid Imaging Reporting and Data System for thyroid nodules: Diagnostic performance and unnecessary biopsy rate. Thyroid.

[37] Zhou S.C., Liu T.T., Zhou J., Huang Y.X., Guo Y., Yu J.H., Wang Y.Y., Chang C. (2020). An ultrasound radiomics nomogram for preoperative prediction of central neck lymph node metastasis in papillary thyroid carcinoma. Front. Oncol..

[38] Dasgupta A., Fatima K., DiCenzo D., Bhardwaj D., Quiaoit K., Saifuddin M., Karam I., Poon I., Husain Z., Tran W.T. (2020). Quantitative ultrasound radiomics in predicting recurrence for patients with node-positive head-neck squamous cell carcinoma treated with radical radiotherapy. Cancer Med..

[39] Fatima K., Dasgupta A., DiCenzo D., Kolios C., Quiaoit K., Saifuddin M., Sandhu M., Bhardwaj D., Karam I., Poon I. (2021). Ultrasound delta-radiomics during radiotherapy to predict recurrence in patients with head and neck squamous cell carcinoma. Clin. Transl. Radiat. Oncol..

[40] Osapoetra L.O., Dasgupta A., DiCenzo D., Fatima K., Quiaoit K., Saifuddin M., Karam I., Poon I., Husain Z., Tran W.T. (2021). Assessment of clinical radiosensitivity in patients with head-neck squamous cell carcinoma from pre-treatment quantitative ultrasound radiomics. Sci. Rep..

[41] Park V.Y., Han K., Lee E., Kim E.K., Moon H.J., Yoon J.H., Kwak J.Y. (2019). Association between radiomics signature and disease-free survival in conventional papillary thyroid carcinoma. Sci. Rep..

[42] Tran W.T., Suraweera H., Quaioit K., Cardenas D., Leong K.X., Karam I., Poon I., Jang D., Sannachi L., Gangeh M. (2019). Predictive quantitative ultrasound radiomic markers associated with treatment response in head and neck cancer. Future Sci. OA.

[43] Tran W.T., Suraweera H., Quiaoit K., DiCenzo D., Fatima K., Jang D., Bhardwaj D., Kolios C., Karam I., Poon I. (2020). Quantitative ultrasound delta-radiomics during radiotherapy for monitoring treatment responses in head and neck malignancies. Future Sci. OA.

[44] Yang X., Tridandapani S., Beitler J.J., Yu D.S., Yoshida E.J., Curran W.J., Liu T. (2012). Ultrasound GLCM texture analysis of radiation-induced parotid-gland injury in head-and-neck cancer radiotherapy: An in vivo study of late toxicity. Med. Phys..

[45] Yang X., Tridandapani S., Beitler J.J., Yu D.S., Yoshida E.J., Curran W.J., Liu T. (2012). Ultrasound histogram assessment of parotid gland injury following head-and-neck radiotherapy: A feasibility study. Ultrasound Med. Biol..

[46] Yang X., Tridandapani S., Beitler J.J., Yu D.S., Chen Z., Kim S., Bruner D.W., Curran W.J., Liu T. (2014). Diagnostic accuracy of ultrasonic histogram features to evaluate radiation toxicity of the parotid glands: A clinical study of xerostomia following head-and-neck cancer radiotherapy. Acad. Radiol..

[47] Meccariello G., Maniaci A., Bianchi G., Cammaroto G., Iannella G., Catalano A., Sgarzani R., De Vito A., Capaccio P., Pelucchi S. (2021). Neck dissection and trans oral robotic surgery for oropharyngeal squamous cell carcinoma. Auris Nasus Larynx.

[48] Van Griethuysen J.J.M., Fedorov A., Parmar C., Hosny A., Aucoin N., Narayan V., Beets-Tan R.G.H., Fillion-Robin J.C., Pieper S., Aerts H.J.W.L. (2017). Computational radiomics system to decode the radiographic phenotype. Cancer Res..

[49] Jensen S.B., Vissink A., Limesand K.H., Reyland M.E. (2019). Salivary Gland Hypofunction and Xerostomia in Head and Neck Radiation Patients. J. Natl. Cancer Inst. Monogr..

[50] Kocher M. (2020). Artificial intelligence and radiomics for radiation oncology. Strahlenther Onkol..

[51] Valdora F., Houssami N., Rossi F., Calabrese M., Tagliafico A.S. (2018). Rapid review: Radiomics and breast cancer. Breast Cancer Res. Treat..

[52] Granzier R.W.Y., van Nijnatten T.J.A., Woodruff H.C., Smidt M.L., Lobbes M.B.I. (2019). Exploring breast cancer response prediction to neoadjuvant systemic therapy using MRI-based radiomics: A systematic review. Eur. J. Radiol..

[53] Chetan M.R., Gleeson F.V. (2021). Radiomics in predicting treatment response in non-small-cell lung cancer: Current status, challenges and future perspectives. Eur. Radiol..

[54] Zwanenburg A., Vallières M., Abdalah M.A., Aerts H.J.W.L., Andrearczyk V., Apte A., Ashrafinia S., Bakas S., Beukinga R.J., Boellaard R. (2020). The Image Biomarker Standardization Initiative: Standardized Quantitative Radiomics for High-Throughput Image-based Phenotyping. Radiology.

[55] Ibrahim H., Liu X., Rivera S.C., Moher D., Chan A.W., Sydes M.R., Calvert M.J., Denniston A.K. (2021). Reporting guidelines for clinical trials of artificial intelligence interventions: The SPIRIT-AI and CONSORT-AI guidelines. Trials.

[56] Coppola F., Faggioni L., Regge D., Giovagnoni A., Golfieri R., Bibbolino C., Miele V., Neri E., Grassi R. (2021). Artificial intelligence: Radiologists’ expectations and opinions gleaned from a nationwide online survey. Radiol. Med..

[57] Chen Y., Stavropoulou C., Narasinkan R., Baker A., Scarbrough H. (2021). Professionals’ responses to the introduction of AI innovations in radiology and their implications for future adoption: A qualitative study. BMC Health Serv. Res..

[58] Huisman M., Ranschaert E., Parker W., Mastrodicasa D., Koci M., Pinto de Santos D., Coppola F., Morozov S., Zins M., Bohyn C. (2021). An international survey on AI in radiology in 1,041 radiologists and radiology residents part 1: Fear of replacement, knowledge, and attitude. Eur. Radiol..

[59] Kang J., Thompson R.F., Aneja S., Lehman C., Trister A., Zou J., Obcemea C., El Naqa I. (2021). National Cancer Institute Workshop on Artificial Intelligence in Radiation Oncology: Training the Next Generation. Pract. Radiat. Oncol..

[60] Wong K., Gallant F., Szumacher E. (2021). Perceptions of Canadian radiation oncologists, radiation physicists, radiation therapists and radiation trainees about the impact of artificial intelligence in radiation oncology-national survey. J. Med. Imaging Radiat. Sci..

[61] Van Hoek J., Huber A., Leichtle A., Härmä K., Hilt D., von Tengg-Kobligk H., Heverhagen J., Poellinger A. (2019). A survey on the future of radiology among radiologists, medical students and surgeons: Students and surgeons tend to be more skeptical about artificial intelligence and radiologists may fear that other disciplines take over. Eur. J. Radiol..

[62] Nagendran M., Chen Y., Lovejoy C.A., Gordon A.C., Komorowski M., Harvey H., Topol E.J., Ioannidis J.P.A., Collins G.S., Maruthappu M. (2020). Artificial intelligence versus clinicians: Systematic review of design, reporting standards, and claims of deep learning studies. BMJ.

