# Supplementary material for: Application of Machine Learning Methods to Improve the Performance of Ultrasound in Head and Neck Oncology: A Literature Review
Source: Cancers (Basel). 2022 Jan 28;14(3):665. doi: 10.3390/cancers14030665 (PMC8833587; doi:10.3390/cancers14030665)
Supplement: Supplementary file 1 [file cancers-14-00665-s001.zip › cancers-1541409-supplementary.pdf]

Supplementary Table S1: Radiomics Quality Score

Authors	Radiomics quality score (RQS; 36=100%)
Acharya et al. 2012	25%
Ardakani et al. 2018 (J Ultrasound Med)	31%
Ardakani et al. 2018 (Eur Radiol)	28%
Bhatia et al. 2016	6%
Chang et al. 2016	19%
Chen et al. 2020	25%
Dasgupta et al. 2020	44%
Ding et al. 2012	0%
Fatima et al. 2021	44%
Galimzianova et al. 2020	15%
Jiang et al. 2020	50%
Kim et al. 2015	25%
Kim et al. 2017	14%
Kwon et al. 2020	36%
Li et al. 2020	31%
Liang et al. 2018	42%
Liu et al. 2018	25%
Liu et al. 2019	33%
Nam et al. 2016	17%
Osapoetra et al. 2021	47%
Park et al. 2019	31%
Park et al. 2020	19%
Park et al. 2021	31%
Prochazka et al. 2019	0%
Raghavendra et al. 2017	0%
Tran et al. 2019	28%
Tran et al. 2020	28%
Tong et al. 2020	39%
Yang et al. 2012 (Med Phys)	19%
Yang et al. 2012 (Ultrasound Med Biol)	25%
Yang et al. 2014	28%
Yoon et al. 2021	31%
Zhao et al. 2020	25%
Zhou et al. 2020	39%
